# An Overview of Cone-Beam Computed Tomography and Dental Panoramic Radiography in Dentistry in the Community

**DOI:** 10.3390/tomography10080092

**Published:** 2024-08-07

**Authors:** David MacDonald, Vera Telyakova

**Affiliations:** Division of Oral & Maxillofacial Radiology, University of British Columbia, Vancouver, BC V6T 1Z3, Canada; vera56@dentistry.ubc.ca

**Keywords:** tomography, dental, panoramic, cone-beam computed tomography

## Abstract

This study reviews the two most important and frequently used systems of tomography used in dentistry today. These are the dental panoramic radiograph (DPR) and cone-beam computed tomography (CBCT). The importance of the DPR has been accentuated by the recent COVID-19 pandemic, as it does not produce an aerosol. Its clinical importance is derived from its panoramic display of the jaws and associated structures and should be examined for incidental findings that may portend a potentially serious outcome. An important recent spin-off of the DPR is the extra-oral bitewing, which can replace its traditional, uncomfortable and aerosol-generating intra-oral counterpart. Although much has been written about them, this paper reviews their essential attributes and limitations in clinical dentistry. Although attempts have been made to reproduce some of the attributes of CT in CBCT such as Hounsfield Units (HU) and improve the contrast resolution of the soft tissues, these remain elusive. Nevertheless, CBCT’s dataset should be appropriately reconstructed to fully display the clinical feature prompting its prescription. In certain cases, more than one mode of reconstruction is required.

## 1. Background

Tomography in dentistry has a long history but is best represented by the dental panoramic radiograph (DPR) and come-beam computed tomography (CBCT). Both are extra-oral, as in each case, the image detector and the X-ray source are positioned outside the patient’s mouth. Before discussing the merits of the DPR and CBCT, it is important to first review why radiography became important in dentistry. Ionizing radiation has played a pivotal role in dentistry by enhancing the quality of both diagnosis and treatment planning [1]. As there have been many reviews on the physics of these technologies, this review will instead focus on their clinical applications in order to inform and inspire dentists, related medical specialties, medical physicists and engineers to better understand how these modalities could be further developed to improve healthcare.

The publication of Roentgen’s radiograph of his wife’s hand was released on the 8 November 1895 [2], only to be followed almost immediately by Walkoff, a dentist, having radiographs taken of his own teeth [3]. In 1913, Raper published perhaps the first book on dental radiography [4]. Why this immediate interest in radiology? Hitherto, dentists, like medical doctors, could evaluate lesions only by manual examination. While the doctor was aided in this by the fact that most of the body is not encased in a solid skeleton, dental examinations were limited by the encasement of lesions within solid teeth and jawbones. Also, dentistry was not then preoccupied with the preservation and augmentation of the ‘smile’ as it is today, but rather with the detection and ablation of potentially serious infections and tumors, hence the ‘surgeon’ in ‘dental surgeon.’ Due to the curvature of the jaws, large, extra-orally positioned X-ray plates gave limited information. These were quickly accompanied by smaller plates that were placed in the mouth, displaying the whole crown to root apex length of the teeth, with the aim of revealing the condition of the tissues around the apices of these teeth. These ‘periapical’ radiographs were shortly joined in 1925 by the bitewings, revealing the extent of dental caries. These bitewings, created by Raper and KODAK [4], completed what in the English-speaking world is known as the ‘full-mouth survey’ (FMS) and in most European countries as the ‘Status’. The FMS became the mainstay of dental imaging, which held sway until the onset of the COVID-19 pandemic, which revealed all intra-oral procedures to be aerosol-generating procedures (AGPs) [5,6] and, therefore, to be avoided wholly or wherever possible, depending on the jurisdiction. Dental caries before WWII was largely seen by many dentists as being an unrelentingly progressive process, despite the fact that even though GV Black, the creator of Black’s cavity design, long foresaw caries as susceptible to prevention [7,8]. In the immediate aftermath of WWII, the discovery of the Stephan curve, revealed that the progress of caries could be reversed by changes in diet [9], which was reinforced by the dramatic drop in caries due to wartime rationing, particularly among children. In parallel to the use of the aforementioned orthogonal radiography in both dentistry and medicine were persistent attempts to develop tomography.

## 2. Dental Panoramic Radiography

**Figure 1:**
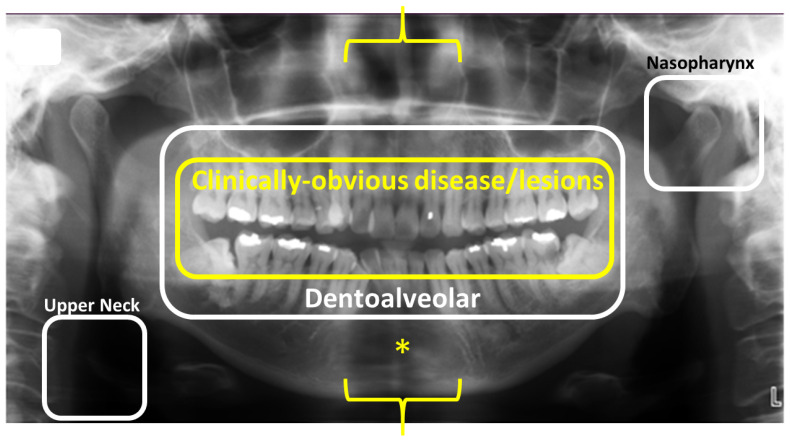
The focus of most dentists when using the dental panoramic radiograph is the dentoalveolar apparatus, the contents of the white box, whereas the yellow box denotes only those structures available to direct dental clinical examination. The areas on the DPR where one would look for nasopharyngeal lesions or calcified carotid artery calcification in the upper neck are indicated by white squares. The yellow brackets denote the anterior sextant, the display of which on the DPR is compromised by both the superimposition of the vertebral column and the narrow focal trough anteriorly. The yellow asterisk is in the center of a ‘radiolucency’, appearing like a cyst or neoplasm, which is an artifact derived from a combination of the vertebral superimposition and narrow focal trough adapted from Figure 9.1 in Reference [12]; MacDonald, Wiley-Blackwell 2020.

The DPR, which combined scanography and tomography, was created by Paatero in the 1950s, making its clinical debut globally in 1960s [10,11]. As it displayed the entire jaws, it was quickly utilized widely, first in dental schools and then in dental offices. The DPR’s lower cost and radiation dose placed it in the front line of dental investigation, particularly for patients who were new to a dental office and/or whose clinical examination revealed at least the suspicion of other disease, for example impacted (and missing) teeth and dysplasias, cysts and neoplasms. Although useful for overviewing the jaws, it did not threaten the FMS, which continued to hold sway because of its superior spatial resolution (image detail). Although the spatial resolution was poorer than that of the intra-oral radiograph [12], the DPR excelled in the display of the whole jaws from condyle to condyle and from the lower orbit to the upper neck (Figure 1). It fully complemented the standard dental examination by displaying structures and lesions in the jaws that could not be assessed either partially or completely by any other means at the time. 

Although the radiation dose imparted to the patient during dental radiography was minute in comparison to that of medical imaging, the Beir report [13], arising out of a study of the Nagasaki and Hiroshima survivors, indicated that even small doses were associated with soft-tissue tumors. The resultant as-low-as-reasonably-achievable (ALARA) led to the need both to justify each exposure and also to ensure that its technique and exposure were optimized [14]. The latter fitted easily into dentistry, where any technique that was performed less than perfectly was professionally abhorrent. Through the 1970s and 1980s, the advent of faster analog (film) and technical advances throughout healthcare was accompanied by the introduction of new technologies in medicine, such as computed tomography (CT) and magnetic resonance (MR) and solid-state conventional imaging. The last entered dentistry as a charge-coupled device (CCD) called RadioVisioGraphy (RVG), developed by Mouyen [15]. Shortly after this, photostimulable phosphors (PSP) were introduced and adapted previous analog DPRs to digital use. Both allowed their images to be integrated into the increasing use of electronic patient records. Although today almost all DPRs are solid state (CCD or CMOS), the largest consecutive series study on digital DPRs, 6252 DPRs, used PSPs [16].

**Figure 2:**
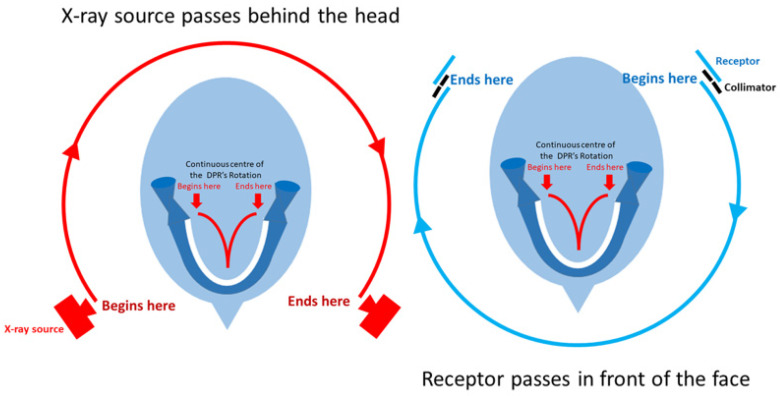
A schematic diagram of the separate paths of rotations of the X-ray source and receptor around the patient’s head. Note the moving center of rotation (red ‘v’) of the modern unit. The receptor in modern units is solid-state, mainly charge-coupled devices (CCD).

DPR technology developed to better reflect the catenary-shaped dental arch [17], transitioning from a single center of rotation, to two, then three and finally, a continuously moving center (Figure 2) and with multiple arch shapes that the operator could choose from, reflecting that of the actual patient. This similarity to the dental arch is actually quite deceptive, as while both the lateral-most thirds faithfully display the posterior sextants, the anterior third, displaying the anterior sextant, is less than perfectly represented due to the superimposition of the vertebral column and its narrower focal trough. The last complicates the precise positioning of the patient within the unit. Together, they can produce artifacts that represent lesions (Figure 1). While these and the strategies to avoid them are taught in dental schools, older clinicians and their assistants are less aware of them. Nevertheless, the maxillary canines, which are at the very junction of the posterior and anterior sextants, can be reliably displayed on the DPR [18].

**Figure 3:**
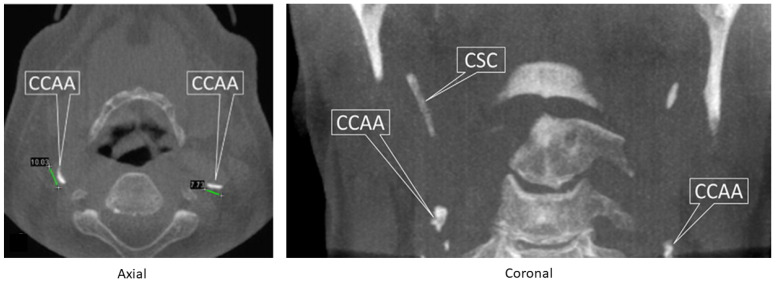
A large field-of-view (FOV) cone-beam computed tomography (CBCT) displaying calcified carotid artery atheromas (CCAA) in the axial and coronal reconstructions (2 different patients). CSC, calcified stylohyoid chain; a normal variant. Adapted from Figures 12.14 and 12.15 in reference [12]; MacDonald, Wiley-Blackwell 2020.

The spatial resolution of the DPR improved to such a degree that an attempt to reduce the radiation dose of the full DPR evolved into the use of segmental DPRs in the early 1990s, which focused only on a specific region of the jaws. While this development detracted from the DPR’s main advantage of allowing the review of the entire jaws from jaw joint to jaw joint, at the same time, attention was drawn to an important finding, the calcified carotid artery atheroma (CCAA), which represents atherosclerosis [19]. As atherosclerosis is a general disease, finding it in one part of the body indicates that it is present elsewhere, such as the coronary arteries. CCAAs, if severe, can also result in strokes, most usually by stenosis and less commonly by embolism. Nevertheless, the incidental finding of possible CCAAs should be excluded from the many other causes of calcifications in the same part of the upper neck, prior to referral to the patients’ primary care physician so as to avoid overloading the system with false positives [19]. CBCT may assist in difficult cases (Figure 3).

While the DPR is better in the detection of lesions in the posterior sextant, it is severely compromised with regard to the maxillary sinus due to its complex anatomy and the limited width of the DPR’s focal trough. In such situations, cross-sectional imaging is necessary [20]. Until the advent of CBCT, due to the difficulty in accessing hospital-based CT and MR (depending on the jurisdiction), the dentist’s only other recourse was the less effective complex-motion tomography, which is now obsolete. 

## 3. Cone-Beam Computed Tomography

**Figure 4:**
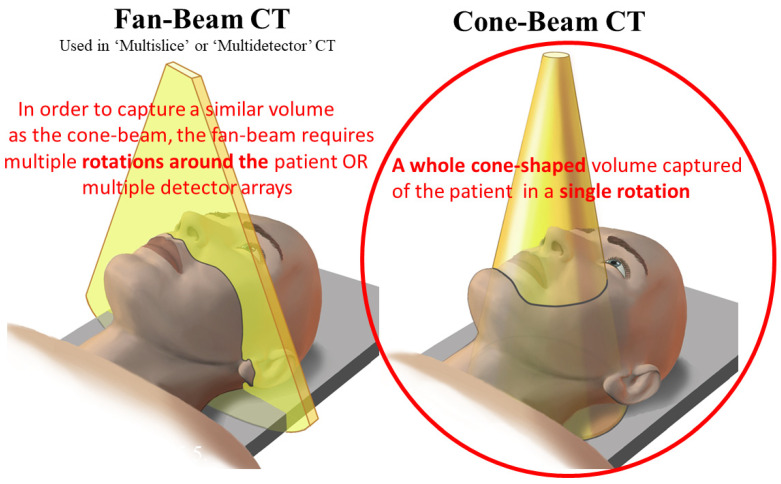
Schematic diagrams comparing fan-beam and cone-beam CT. The red circle denotes the single 360° rotation (or less for most units) of the CBCT. Adapted from Figure 5.5 in reference [12]; MacDonald, Wiley-Blackwell 2020.

CBCT, the sixth generation of CT technology, prior to its clinical debut in dentistry, was used in emergency rooms (ER) and operating rooms (OR) with a C-arm [21]. When applied solely to dentistry, it was called ‘dental CBCT’ [22]. This dental CBCT was created separately in Italy [23] and Japan [24]. The term cone-beam reflects the beam’s conical shape (which can also be rectangular) rather than the fan-shaped beam of medical CT (Figure 4). CBCT uses only one 360° rotation or less in comparison to CT, which uses multiple rotations, based on its spiral CT base [25]. Currently, there are at least 50 manufacturers of CBCT globally [26].

The earliest dental CBCT units had the patients lying supine. These were quickly replaced by the current units that use a vertical DPR-like gantry, in which the patients are either seated or stood [23]. As this requires only a small footprint, it is ideal for the dental office, where space is a premium. Although CBCT does not require the high-tension power supply and floor strengthening required for medical CT, it does require shielding to protect the adjacent operatories from both the primary and scattered radiation. 

CBCT units use ‘flat panel detectors’ (FPD) constructed of amorphous silicon, rather than the image intensifiers frequently used in CT. FPD have the advantage of not producing the geometric distortion that occurs with image intensifiers. In addition, FPD have a wider dynamic range, better signal-to-noise ratio (SNR) and better resolution [27]. 

**Figure 5:**
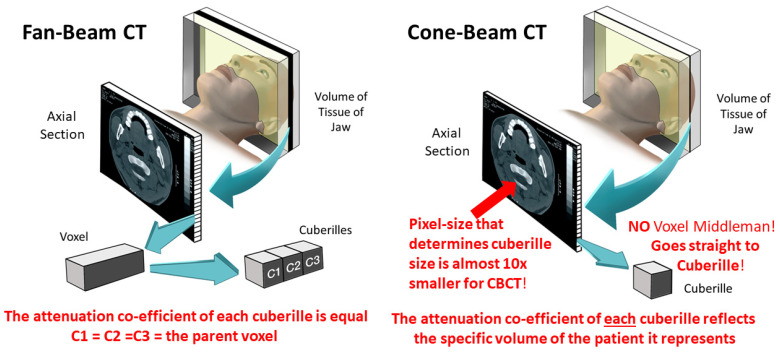
Schematic diagrams comparing the construction of cuberilles. The cone-beam diagram indicates that the pixels are smaller, almost 10 times smaller than those for most fan beams. Adapted from Figures 4.9 and 5.6 in Reference [12]; MacDonald, Wiley-Blackwell 2020.

Initially, the field-of-view (FOV) included almost the whole head. While this far exceeded the region-of-interest (ROI) of the jaws, there was little anxiety initially of over-radiation, as the majority of patients were mature, substantially or wholly edentulous adults who were being evaluated for osseo-integrated implants [28]. This changed when CBCT cephalometry of the orthodontic patient became popular, as the majority were children and most susceptible to radiation-induced damage. Nevertheless, CBCT is preferred over medical CT for the cleft-lip and palate and craniofacial anomality of the child patient, as it imparts a much lower radiation dose. This is particularly important for those patients who require multiple surgeries, each needing cross-sectional imaging of the facial skeleton. CBCT reconstructs 3D images by generating cuberilles directly, each with its own attenuation coefficient (Figure 5). This allows 3D reconstructions with better spatial resolution in the Z plane (patient’s long axis), in addition to the axial (XY) plane. In addition, CBCT’s spatial resolution can be as good as 0.076 mm^2^ in contrast to the 0.25 mm^2^ of the ultra-high-resolution CT (UHRCT), currently the best CT, for cardiac imaging [29]. 

**Figure 6:**
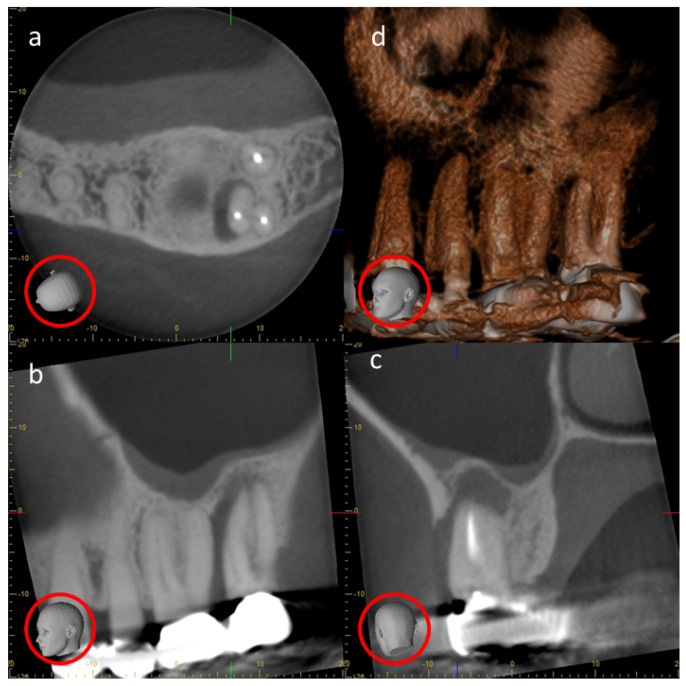
Depiction of a 4 cm × 4 cm field-of-view (FOV) cone-beam computed tomography (CBCT) of a periapical–periodontal lesion on an already-root-treated molar tooth. This is an orthogonal, multiplanar reconstruction: (**a**) is axial, (**b**) is sagittal and (**c**) is coronal. The ‘heads’ indicated by the red circles clarify their orientation. (**d**) is a 3D reconstruction of the whole 4 × 4 dataset. (**a**–**c**) are Adapted from Figure 3, I Reference [6] MacDonald, D.: Reitzik, S. Int Dent J 2022, 72, 448-55.

**Figure 7:**
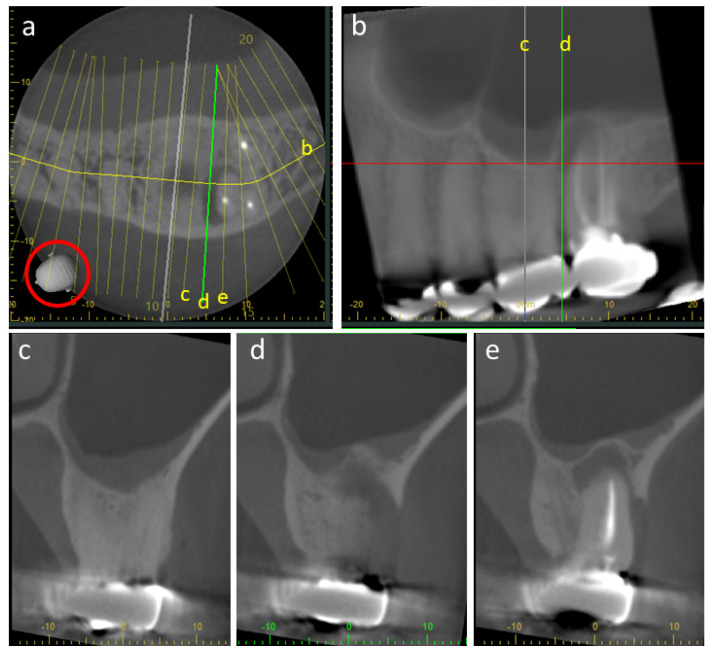
Depicted is the same 4 cm × 4 cm FOV CBCT dataset as in Figure 6 but displayed in a curved/panoramic multiplanar reconstruction: (**a**) is axial; (**b**) is panoramic (the white line in (**a**)) and (**c**–**e**) are transaxial; and (**d**) is the green line in (**a**), which are immediately before (**c**) and behind (**e**). The ‘head’ displays the orientation they are all constructed from.

CBCT’s lower radiation dose, smaller footprint and better accessibility and spatial resolution than CT saw it increasingly applied to almost every aspect of dentistry, even to those disciplines that hitherto were adequately served by conventional radiography alone. When CBCT is used for endodontics (root-canal treatment), a better spatial resolution was required to reveal the finely detailed and complex pulp–canal network. The advantage of CBCT over traditional radiography was revealed in hitherto the largest dental consecutive case series [30], applying Fryback and Thornbury’s ‘Hierarchy for efficacy’ (developed for medical radiology) [31]. This reported CBCT to be superior to conventional periapical radiographs for most features, particularly with regard to the hierarchy’s higher levels of ‘therapeutic impact’ and ‘diagnostic impact’ [30]. CBCT better displayed missed canals, particularly in already-treated teeth, facilitating their effective re-treatment [30]. Unfortunately, it was less effective in revealing external root reception, an important diagnostic and prognostic feature. External root reception was also an important diagnostic and prognostic feature in orthodontics, particularly with regard to impacted maxillary canine teeth. Each unerupted tooth is directed to its final place in the dental arch by a gubernaculum [32], a soft-tissue tract leading from its crown. As the maxillary canine has the longest eruption pathway, it may not reach its proper place in the dental arch as a fully erupted tooth, but instead, it may be impacted against the roots of adjacent lateral incisors, causing their resorption. Failure to dis-impact the canines in time results in the lateral incisor’s loss, resulting in orthodontic and cosmetic problems. The CBCT of such canines can reduce the treatment time by up to 73% [33]. When both orthogonal (Figure 6) and curved/panoramic (Figure 7) multiplanar reconstructions (MPRs) constructed from the same dataset are reviewed, such root resorption can be identified rather than by either alone [34]. Although the use of one or the other is broadly adequate for the other features of the impacted tooth, it is recommended that both are used. This makes excellent sense, as the dataset has already been made, and the patient suffers no further radiation exposure. 

In addition to implant planning and to detecting all the lesions that could not be identified by the DPR, such as dysplasias, cysts and neoplasms (odontogenic and malignant) [12], CBCT allows for the identification of the superior maxillary artery. Although its violation, usually in the course of implant surgery, is not life-threatening, its haemostasis is difficult [35].

A further aid to the improved detail of the image is to reduce patient movement as much as possible to avoid unsharpness [22]. The use of a chair rather than the patient standing greatly assists, particularly if the patient is challenged by medical conditions that affect stability. Another source of movement is the very CBCT unit itself, particularly when a DPR-like gantry has been used, in which the rotating X-ray source and image receptor unit is cantilevered off a single stanchion. The oscitations produced when such units rotate also causes unsharpness. Some units circumvent this problem by having two stanchions between which the X-ray source and image receptor unit rotate.

## 4. Disadvantages of Cone-Beam Computed Tomography

### 4.1. Radiation Dose

Before considering CBCT specifically, we should develop our earlier discourse on radiation dose in dentistry. As “the risk of a dental radiographic procedure has been estimated at approximately one-in-a-million for causing a fatal malignancy”, there is no known safe level of radiation dose. When the difference between that of a DPR and an FMS were compared logarithmically, the FMS imparted the higher radiation dose [36]. Furthermore, while most modern dental radiographic technologies individually impart significantly lower radiation doses than expected in medicine, the global dose is high due to the fact that particularly in the West, a substantial proportion of the population visit the dentist regularly. In Canada, just before the COVID-19 pandemic, three-quarters of the population visited their dentist at least once that year [37]. Although due to COVID-19, this has declined, it is still substantial at almost two-thirds [38]. This means that the efficacy of systems of work that were created a century ago should now be reviewed. Farman first suggested that consideration be given to replacing the FMS with the DPR over two decades ago [39]. Therefore, upon completion of a full clinical examination that indicates a general radiological survey of the patient, particularly of the new patient, replacing the traditional FMS with a DPR and perhaps bitewings will reduce the radiation dose to a third. While a study to evaluate this should be first performed, the recent COVID-19 pandemic’s revelation that an intra-oral radiograph generated an aerosol [5,6] and the WHO’s admonition at the declaration of the pandemic’s end last year not to abandon the systems that brought it under control [40] have advanced the central role that (extra-oral) tomography can play in dentistry going forward. This not only affects CBCT and the DPR, but also a DPR spin-off, the extra-oral bitewing (EBW). 

The reduction in the radiation dose to the dental patient is an overwhelming consideration, as most dental patients, unlike medical patients, are not being investigated for a potentially life-threatening illness. The most effective method of reducing radiation dose is using imaging only when it is necessary. It should never be used unthinkingly routinely, nor merely to satisfy professional curiosity. This is particularly more so for children, who are both more susceptible to radiation-induced damage [41] and also have their whole lives ahead of them to manifest it. Furthermore, while ‘effective dose’ is the sum of all tissue-weighted tissues, it does not consider age or sex and, therefore, can be used only at a population level and not at the individual level [41]. The need to reduce the radiation dose is upheld by local, national and regional jurisdictions; examples are the HARP Act for Ontario [42], Safety Code 30 for Canada [43], IR(ME)R for the United Kingdom [44] and, specifically for CBCT, SEDENTEXCT for the European Union [45].

CBCT’s highly acclaimed lower radiation dose in comparison to CT arises mainly from the fact that only one rotation, if that, of the patient is required, whereas CT required multiple rotations. This reduction in radiation imparted also affects the quality of the images. CBCT’s poor SNR results from the low number of photons used to generate the image. A further cause of noise intrinsic to CBCT is scatter, because the cone-shaped beam interrogates a large block volume of tissue in comparison to the thinner slices of CT. The use of smaller FOVs improves both the SNR and spatial resolution. The small FOV overall reduces the radiation dose delivered and is one of the reasons why some jurisdictions restrict the use of CBCT only for dentoalveolar (teeth and jaws) purposes and the FOV size to no more than 8 cm × 8 cm [46]. Furthermore, a small FOV can be used with better spatial resolution where fine detail is a priority, such as in endodontics (Figure 6 and Figure 7).

### 4.2. Metal Artifacts and Use of Metal Artifact Reduction Software

**Figure 8:**
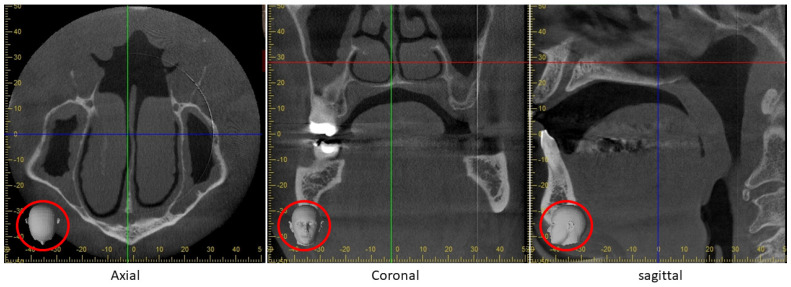
A large FOV CBCT, which includes not only the whole jaws, but also the clivus, most of the antro-nasal complex, the vertebral column and the nasopharynx. The standard orthogonal planes are displayed. They are also represented by the red-ringed heads. The positions of these planes are axial (red line), coronal (blue line) and sagittal (green line). Note also the metal of the dental restorations attenuates the primary beam at the level of the occlusal plane (coronal and sagittal planes). This artifact extends through the whole thickness of the head at that level, including the odontoid process (dens) of the second cervical vertebra (just below the anterior arch of the first cervical vertebra).

CBCT images, like those of CT, are affected by metal dental restorations, as the jaws in many patients, particularly mature adults, will have large amalgam ‘fillings’, fixed crowns, bridges and implants. These artifacts present scatter (white streaks) and beam-hardening (black bands). Unfortunately, the whole of that horizontal plane, which is consistent with the occlusal plane, is disrupted (center and right in Figure 8). Figure 8 (right) also displays image disruption of the base of the odontoid process (dens) of the second cervical vertebra. While metal artifact reduction (MAR) software has a long history in medicine, many radiologists do not use it as there is a real concern that such reconstructed images may obscure real pathology. Therefore, when faced by the disruption displayed in Figure 8, most radiologists will rescan behind the teeth after altering the angulation of the patient’s head. However, such artifacts and the use of MAR make little difference in dentistry, since the presence of disease can be excluded in most cases by a prior clinical examination and conventional radiography such as the DPR. Nevertheless, the impact of various MAR software used with CBCT varies with the type of restoration and imaging mode [47]. 

### 4.3. Potential Medico-Legal Complcations

Recently, a conventional dental radiograph inadequately displayed the relationship of a tooth to the mandibular canal, which contains the nerve supplying sensation to the lower lip. Its violation by the removal of that tooth caused permanent lip numbness and resulted in a medico-legal case [48], which serves as a salutary warning for similar mishaps with CBCT [49]. Therefore, the FOV chosen for a particular patient should reflect the size of the area of the clinical ROI. A small FOV is appropriate for one or two adjacent teeth, such as in endodontics, impacted canines or a single implant, whereas multiple implants may require a medium FOV (8 cm × 8 cm). These small and medium FOVs satisfy most other dental indications. As they encompass only the jaws, the dentist’s primary area of expertise, there is no pressing need to secure the assistance of a radiologist. This changes if larger FOVs are used, which include the orbit, the base of the skull and the vertebral column (Figure 8), which are properly the remit of the medical doctor. While we have already covered this with regard to CCAAs, there are other lesions. While orthodontists have for a long time used lateral cephalograms (a standardized projection of the lateral skull and jaws) in the standard primary investigation of their patients, the vast majority who are overtly healthy, a recent large study revealed that almost a fifth of such images included overlooked incidental findings, some of which could potentially affect the treatment plan [50]. 

It is a medico-legal requirement that clinicians should be able to evaluate everything that is displayed on the CBCT image, even if it extends outside the usual area of their dental interest. Most dentists, except for the oral and maxillofacial specialties, do not have the training or experience to report on these regions. 

### 4.4. Clinically Important Aspects Peculiar to CBCT in a Dental Context

Although CBCT captures the desired volume of the patient in a single rotation or less with most modern units (Figure 4) and, thereby, with a reduced radiation dose and with a much better spatial resolution and in all directions than CT (Figure 5), the contrast resolution is poorer. Although the last has improved over the last decades, despite repeated attempts, no Hounsfield Units (HU) can be meaningfully calculated for CBCT, as not only is “there a difference in the values of HU …recorded under the same condition on CT and CBCT” [51], but also, in another study, which resorted to using gray values (GVs), a pseudo-HU scale reported that the lack of GV standardization is a major problem for most CBCT devices [52]. These recent studies, along with many others addressing this topic, have been in vitro rather than in vivo, which indicates that more work is required before the clinical utility of these parameters can be properly assessed.

DPR and CBCT are effective in addressing almost all the needs of general and specialist dentists, primarily because they display mineralized tissue, bone, teeth, radiopaque lesions within the jaws due to neoplastic or dysplastic bone or radiopaque lesions within the soft tissues, such as the aforementioned CCAAs [12]. But when the lesion arises either within soft tissue or extends into it from the jawbone, both modalities are ineffective, because their contrast resolution is inadequate. Ultimately, all lesions with potentially serious outcomes should be timely referred to medical radiological centers for CT, MR or fused positron-emission tomography with CT (PET-CT) [12].

## 5. Dental Panoramic Radiography—Revisited

Quietly in the background of this CBCT revolution, another was occurring with the DPR. The transition from three centers of rotation to a continuously moving center of rotation (Figure 4) was not only completed but also continued to evolve with software that allowed the clinician to select an arch shape that best suited the patient. In the 1990s, it quickly moved from using analog (film) to digital receptors (terms ‘detectors’ or ‘sensors’ are also used). Initially, the digital systems were either PSP or solid-state detectors; the latter later overwhelmed the market [53]. This digitalization of dental images used in conjunction with the electronic patient record enhances their security and retrieval. 

Digitization not only allowed these images to be displayed on a monitor at the chairside and its brightness to be adjusted [12] but were also particularly invaluable for the child patient–parent dialog. This has been facilitated by the modern DPR’s range of exposure conditions and trough shapes. The same conditions and trough shape can be used for every child between 6 and 12 years of age (when the dentition is transitioning from baby teeth (deciduous or primary dentition) to completely adult teeth (permanent or secondary dentition). Although the DPR remains the modality of choice for most patients, CBCT is invaluable if the patient has a cleft-lip and/or palate or other craniofacial abnormality [12]. As mentioned earlier, these conditions require several operations, each needing a new presurgical scan. 

### Extra-Oral Bitewing

**Figure 9:**
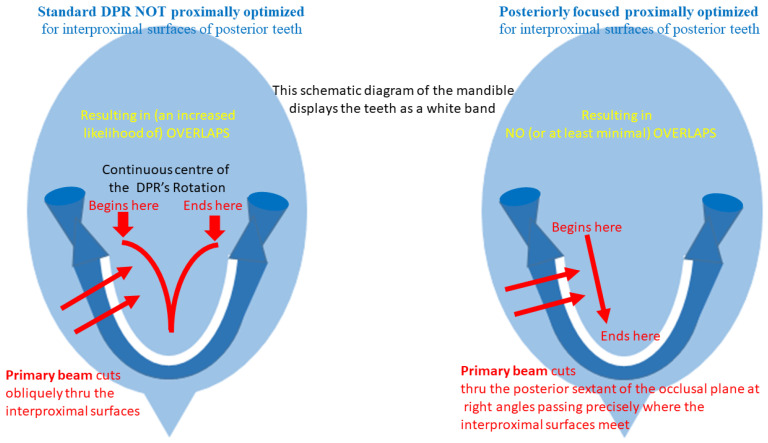
Schematic diagrams indicating the moving center of rotation and the primary rays passing thorough the dental arch compared to that of the same unit using the bitewing software that employs the postero-anterior movement of the center of rotation and the primary rays now passing perpendicularly through the dental arch, which minimizes the overlap between the adjacent proximal surfaces of the posterior (molar and premolar) teeth.

**Figure 10:**
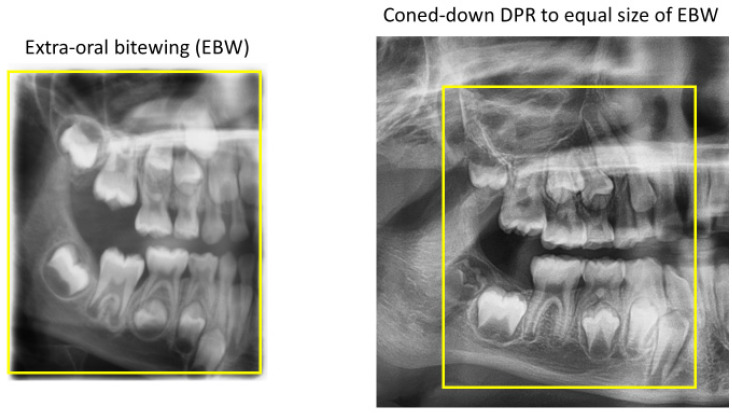
The extra-oral bitewing (EBW) of the posterior deciduous (baby) teeth produced by an EBW program on a DPR unit. The overlap between the adjacent proximal surfaces of the posterior teeth is either absent or minimal compared with the more extensive overlaps between them on the DPR. Additionally, the EBW covers more of the dentoalveolus, exhibiting both erupted and unerupted teeth and their root development. The extent of the EBW relative to the DPR is denoted by the yellow box. These last features are more important to the dentist who cannot otherwise evaluate the primary or mixed dentition of children either clinically or by the smaller, traditional intra-oral bitewings.

The extra-oral bitewing (EBW) is an important spin-off of the DPR, but unlike the DPR, the EBW’s primary rays pass perpendicularly between the posterior teeth (Figure 9), so that overlap between their proximal surfaces is either absent or minimal in comparison to the more extensive overlaps between them on the DPR (Figure 10). The overlapping teeth on the DPR reflect the DPR’s central ray running obliquely through the dental arch (Figure 9). Interproximal caries is now easier to see in the EBW than in the DPR. Additionally, the EBW covers more of the dentoalveolus than the traditional and much smaller intra-oral bitewing, exhibiting the root development of erupted teeth and presence (or absence) of unerupted teeth (Figure 10). These are more important to the dentist who cannot evaluate the child’s primary or mixed dentition either clinically or by the traditional intra-oral bitewings either due to technical difficulty or to the child’s lack of cooperation.

The importance of the EBW has been heightened by the recent COVID-19 pandemic, which reported intra-oral radiographs as AGPs [5,6], particularly among child patients and those patients with strong gag-reflex and prior resistance to intra-oral radiography [5,6]. Although a disadvantage is false positives for caries [5,6,54], this may not be so important, as all radiological findings should be followed by a clinical re-examination of the patient.

## 6. Evaluation of Lesions and Jaw Anatomy

DPR not only is a 2D representation of a 3D structure, in contrast to the cross-sectional display of CBCT, but also, its inherent superimposition of contralateral structures upon the ipsilateral structures detracts from their clinical impact. Furthermore, the DPR’s narrow focal trough, particularly in the anterior sextant, can on occasion display a partial thickness, which creates a radiolucent defect, resembling a lesion, such as a cyst or neoplasm (Figure 1). A further hinderance to interpretation in the DPR’s anterior sextant is the superimposition of the vertebral column (cervical spine), particularly the superimposition of the intervertebral discs, which creates additional artifacts that may also mimic disease.

While both DPRs and CBCTs, as with any imaging modality, must be properly positioned or, in the case of the compromised patient, as optimally positioned as possible, the DPR intrinsically suffers from magnification (about 1.3X; dependent on the make), particularly in the vertical plane, and distortion, particularly in the horizontal plane [12,17]. The former is due to both the upward angulation of the central beam and the distance between the ipsilateral structures and the display of their images on the now contralaterally positioned detector (refer to Figure 2). The distortion, defined as the variable magnification, may vary with the patient and even between two DPRs taken of the same patient. As a result, any measurement made on a DPR, particularly in the horizontal plane, is of very little clinical value. Nevertheless, some indication of the size of a lesion on a DPR can be determined if ‘dental units’ are used [12]. On the other hand, measurements made within the CBCT’s dataset can be safely used, provided a 1 mm interval between the end point of the surgical cut, implant or root-canal filling is respected. Such a precaution avoids the violation of neurovascular structures, such as the mandibular canal, as well as mental, nasopalatine and other foramina. 

In almost all dental CBCT units, the patient is either seated or standing vertically in comparison to their supine position in medical CT units [12,55]. This means that the pharynx in dental CBCT hangs down from the base of the skull like a curtain, with its folds fully developed (Figure 10(left)). Therefore, nasopharyngeal carcinoma, an important malignant tumor which is most prevalent in East Asians and their diasporas, could be visible on a large FOV CBCT (Figure 8) taken for dental purposes [56]. Unfortunately, as many such datasets are not forwarded to the radiologist for evaluation, the opportunity to detect these lesions at an early and more easily treatable stage is lost. This vertical positioning also affects the position of the tongue and soft palate when investigating a patient for obstructive sleep apnea (OSA) [57].

## 7. Artificial Intelligence

Artificial intelligence (AI) has been recruited to assist dentists to identify findings which are symptomless. Such findings are termed incidental findings (IFs) or incidentalomas. Such AI software prompts the dentist to review them clinically and determine whether they require treatment. One important set of IFs are the periapical radiolucencies of inflammatory origin (PRIOs), as their subsequent treatment may not simply address a dental problem but rid the body of a source of inflammation. AI on DPRs and CBCT revealed many such PRIOs [58]. Nevertheless, due to the DPRs’ multiple variables, the development of an effective and reproducible system of Machine Learning for diagnosis requires more work [59]. The performance of a new deep-learning (DL) AI model was the best for implant, crowns, and implant-supported crowns and the worst for caries and dental calculus [60].

Last but not least, periodontal disease, accounting for about half of dental disease, results in the loss of the bone height of the alveolar bone which invests the tooth roots. AI is being prepared for an important role in its assessment [61].

## 8. Conclusions

First, a complete history and a full clinical examination must be performed. From these, the indications for radiography emerge. Symptoms and clinical findings should direct appropriate radiography. No findings on a routine check-up of the patient indicate no need for radiography, unless the patient is new to the office and with no access to previous radiographs. This investigation should be restricted to a DPR and BWs. The DPR may reveal retained roots and also reveal CCAAs. BWs may indicate caries and should prompt a re-examination. Previously treated endodontic cases should include small FOV CBCT. Any maxillary lesion, if not a medical emergency, should indicate CBCT. While CBCT on children should be avoided in principle, if multiple surgeries are contemplated, then CBCT is preferred to medical CT. Extra-oral bitewings can be invaluable for child patients, particularly when they are uncooperative. Intra-oral radiography should be avoided when one is confronted by a potentially infective and lethal aerosol-borne virus. Finally, every DPR and CBCT dataset should be carefully reviewed to ensure that all findings are detected and reported.

## Data Availability

Not applicable.

## References

[1] Wenzel A. (2021). Radiographic modalities for diagnosis of caries in a historical perspective: From film to machine-intelligence supported systems. Dentomaxillofac. Radiol..

[2] Sen M., Honavar S.G. (2021). Wilhelm Conrad Röntgen: Finding X. Indian J. Ophthalmol..

[3] Todd R. (2014). Cone beam computed tomography updated technology for endodontic diagnosis. Dent. Clin. N. Am..

[4] Manson-Hing L.R. (1978). H.R. Raper: Dental radiology pioneer. Oral Surg. Oral Med. Oral Pathol..

[5] MacDonald D.S., Colosi D.C., Mupparapu M., Kumar V., Shintaku W.H., Ahmad M. (2021). Guidelines for oral and maxillofacial imaging: COVID-19 considerations. Oral Surg. Oral Med. Oral Pathol. Oral Radiol..

[6] MacDonald D., Reitzik S. (2022). New normal radiology. Int. Dent. J..

[7] Young D.A., Nový B.B., Zeller G.G., Hale R., Hart T.C., Truelove E.L., American Dental Association Council on Scientific Affairs (2015). The American Dental Association Caries Classification System for clinical practice: A report of the American Dental Association Council on Scientific Affairs. J. Am. Dent. Assoc..

[8] Parameswaran A. (2016). Evolving from principles of GV Black. J. Operative Dent. Endodont..

[9] Bowen W.H. (2013). The Stephan Curve revisited. Odontology.

[10] Tammisalo E.H. (1975). Professor Yrjö V. Paatero—The pioneer of panoramic oral tomography. Dentomaxillofac. Radiol..

[11] Patero Y.V. (1961). Pantomography and orthopantomography. Oral Surg. Oral Med. Oral Pathol..

[12] MacDonald D. (2020). Oral and Maxillofacial Radiology: A Diagnostic Approach.

[13] National Research Council Beir VII: Health Risks from Exposure to Low Levels of Ionizing Radiation: Report in Brief. https://nap.nationalacademies.org/resource/11340/beir_vii_final.pdf.

[14] Farman A.G. (2005). ALARA still applies. Oral Surg. Oral Med. Oral Pathol. Oral Radiol. Endod..

[15] Mouyen F., Benz C., Sonnabend E., Lodter J.P. (1989). Presentation and physical evaluation of RadioVisioGraphy. Oral Surg. Oral Med. Oral Pathol..

[16] MacDonald D., Yu W. (2020). Incidental findings in a consecutive series of digital panoramic radiographs. Imaging Sci. Dent..

[17] Langland O.E., Langlais R.P., McDavid W.D., DelBalso A.M. (1989). Panoramic Radiography.

[18] Abdulkreem A., Bhattacharjee T., Alzaabi H., Alali K., Gonzalez A., Chaudhry J., Prasad S. (2024). Artificial intelligence-based automated preprocessing and classification of impacted maxillary canines in panoramic radiographs. Dentomaxillofac. Radiol..

[19] MacDonald D., Chan A., Harris A., Vertinsky T., Farman A.G., Scarfe W.C. (2012). Diagnosis and management of calcified carotid artery atheroma: Dental perspectives. Oral Surg. Oral Med. Oral Pathol. Oral Radiol..

[20] Constantine S., Clark B., Kiermeier A., Anderson P.P. (2019). Panoramic radiography is of limited value in the evaluation of maxillary sinus disease. Oral Surg. Oral Med. Oral Pathol. Oral Radiol..

[21] Hott J.S., Deshmukh V.R., Klopfenstein J.D., Sonntag V.K., Dickman C.A., Spetzler R.F., Papadopoulos S.M. (2004). Intraoperative Iso-C C-arm navigation in craniospinal surgery: The first 60 cases. Neurosurgery.

[22] Kaasalainen T., Ekholm M., Siiskonen T., Kortesniemi M. (2021). Dental cone beam CT: An updated review. Phys. Med..

[23] Abramovitch K., Rice D.D. (2014). Basic principles of cone beam computed tomography. Dent. Clin. N. Am..

[24] Araki K., Maki K., Seki K., Sakamaki K., Harata Y., Sakaino R., Okano T., Seo K. (2004). Characteristics of a newly developed dentomaxillofacial X-ray cone beam CT scanner (CB MercuRay): System configuration and physical properties. Dentomaxillofac. Radiol..

[25] Kalender W.A. (2000). Computed Tomography: Fundamentals, System Technology, Image Quality, Applications.

[26] Al-Okshi A., Horner K., Rohlin M. (2021). A meta-review of effective doses in dental and maxillofacial cone beam CT using the ROBIS tool. Br. J. Radiol..

[27] Baba R., Ueda K., Okabe M. (2004). Using a flat-panel detector in high resolution cone beam CT for dental imaging. Dentomaxillofac. Radiol..

[28] Bryant S.R., MacDonald-Jankowski D., Kim K. (2007). Does the type of implant prosthesis affect outcomes for the completely edentulous arch?. Int. J. Oral Maxillofac. Implant..

[29] Oostveen L.J., Boedeker K.L., Brink M., Prokop M., de Lange F., Sechopoulos I. (2020). Physical evaluation of an ultra-high-resolution CT scanner. Eur. Radiol..

[30] Bhatt M., Coil J., Chehroudi B., Esteves A., Aleksejuniene J., MacDonald D. (2021). Clinical decision-making and importance of the AAE/AAOMR postion statement for CBCT examination in endodontic cases. Int. Endod. J..

[31] Fryback D.G., Thornbury J.R. (1991). The efficacy of diagnostic imaging. Med. Decis. Making.

[32] Liu P., Li R., Cheng Y., Li B., Wei L., Li W., Guo X., Li H., Wang F. (2024). Morphological variation of gubernacular tracts for permanent mandibular canines in eruption: A three-dimensional analysis. Dentomaxillofac. Radiol..

[33] Schubert M., Proff P., Kirschneck C. (2018). Improved eruption path quantification and treatment time prognosis in alignment of impacted maxillary canines using CBCT imaging. Eur. J. Orthod..

[34] MacDonald D., Alebrahim S., Yen E., Aleksejuniene J. (2023). Cone-beam computed tomographic reconstructions in the evaluation of maxillary impacted canines. Imaging Sci. Dent..

[35] Park J.A., Kim D., Yang S., Kang J.H., Kim J.E., Huh K.H., Lee S.S., Yi W.J., Heo M.S. (2024). Automatic detection of posterior superior alveolar artery in dental cone-beam CT images using a deeply supervised multi-scale 3D network. Dentomaxillofac. Radiol..

[36] Farman A.G., Farman A.G. (2009). Panoramic radiology: Role in ADA/FDA guidelines. Panoramic Radiology.

[37] Statistics Canada Dental Care. 2018. Released on 16 September 2019. https://www150.statcan.gc.ca/n1/pub/82-625-x/2019001/article/00010-eng.htm.

[38] Statistic Canada More than One-Third of Canadians Reported They Had Not Visited a Dental Professional in the Previous 12 Months. 2022. Released on 6 November 2023. https://www150.statcan.gc.ca/n1/daily-quotidien/231106/dq231106a-eng.htm.

[39] Farman A.G. (2002). There are good reasons for selecting panoramic radiography to replace the intraoral full-mouth series. Oral Surg. Oral Med. Oral Pathol. Oral Radiol. Endod..

[40] Burki T. (2023). WHO ends the COVID-19 public health emergency. Lancet Respir. Med..

[41] Pauwels R., Scarfe W.C., Scarfe W.C., Angelopoulos C. (2018). Radiation dose risks, and protection in CBCT. Maxillofacial Cone-Beam Computed Tomography Principles, Practice and Clinical Applications.

[42] Healing Arts Radiation Protection Act. https://www.canlii.org/en/on/laws/stat/rso-1990-c-h2/latest/rso-1990-c-h2.html.

[43] Health Canada (2022). Radiation Protection in Dentistry—Recommended Safety Procedures for the Use of Dental X-Ray Equipment—Safety Code 30. https://www.canada.ca/en/health-canada/services/environmental-workplace-health/reports-publications/radiation/radiation-protection-dentistry-recommended-safety-procedures-use-dental-equipment-safety-code-30.html.

[44] Ionising Radiation (Medical Exposure) Regulations (IR(ME)R). https://www.cqc.org.uk/guidance-providers/ionising-radiation/ionising-radiation-medical-exposure-regulations-irmer.

[45] SEDENTEXCT Guidelines on CBCT for Dental and Maxillofacial Radiology. https://sedentexct.eu/training/index.htm.

[46] Royal College of Dentists of Ontario Standard of Practice. Dental CT Scanners. https://az184419.vo.msecnd.net/rcdso/pdf/standards-of-practice/RCDSO_Standard_of_Practice__Dental_CT_Scanners.pdf.

[47] Kim Y.H., Lee C., Han S.S., Jeon K.J., Choi Y.J., Lee A. (2020). Quantitative analysis of metal artifact reduction using the auto-edge counting method in cone-beam computed tomography. Sci. Rep..

[48] Holsten G., Card G.A., Wu K.L.C. Supreme Court of British Columbia. https://ca.vlex.com/vid/holsten-v-card-681586129.

[49] Friedland B., Scarfe W.C., Scarfe W.C., Angelopoulos C. (2018). Ethical and medicolegal issues related to CBCT. Maxillofacial Cone-Beam Computed Tomography Principles, Practice and Clinical Applications.

[50] MacDonald D., Patel A., Zou B., Yen E., Vora S.R. (2022). A retrospective study of incidental findings occurring in a consecutive case series of lateral cephalograms of 12- to 20-year-old patients referred for routine orthodontic treatment. Imaging Sci. Dent..

[51] Mikic M., Vlahovic Z., Stevanović M., Arsic Z., Mladenovic R. (2022). The importance of correlation between CBCT analysis of bone density and primary stability when choosing the design of dental implants-Ex Vivo study. Tomography.

[52] Pauwels R., Jacobs R., Singer S.R., Mupparapu M. (2015). CBCT-based bone quality assessment: Are Hounsfield units applicable?. Dentomaxillofac. Radiol..

[53] Aps J. (2019). Imaging in Pediatric Dental Practice. A Guide to Equipment, Techniques and Clinical Considerations.

[54] Chan M., Dadul T., Langlais R., Russell D., Ahmad M. (2018). Accuracy of extraoral bite-wing radiography in detecting proximal caries and crestal bone loss. J. Am. Dent. Assoc..

[55] MacDonald D., Angelopoulos C., Scarfe W.C., Scarfe W.C., Angelopoulos C. (2018). Cone-beam computed tomography and maxillofacial diagnosis. Maxillofacial Cone-Beam Computed Tomography Principles, Practice and Clinical Applications.

[56] MacDonald D.S., Martin M.A., Wu J.S. (2024). The responsibility of dentists in radiologic examination of the nasopharynx. Oral Surg. Oral Med. Oral Pathol. Oral Radiol..

[57] Liu C.N., Kang K.T., Yao C.J., Chen Y.J., Lee P.L., Weng W.C., Hsu W.C. (2022). Changes in cone- beam computed tomography pediatric airway measurements after adenotonsillectomy in patients with OSA. JAMA Otolaryngol. Head Neck Surg..

[58] Kazimierczak W., Wajer R., Wajer A., Kiian V., Kloska A., Kazimierczak N., Janiszewska-Olszowska J., Serafin Z. (2024). Periapical lesions in panoramic radiography and CBCT imaging-assessment of AI’s diagnostic accuracy. J. Clin. Med..

[59] Delamare E., Fu X., Huang Z., Kim J. (2024). Panoramic imaging errors in machine learning model development: A systematic review. Dentomaxillofac. Radiol..

[60] Başaran M., Çelik Ö., Bayrakdar I.S., Bilgir E., Orhan K., Odabaş A., Aslan A.F., Jagtap R. (2022). Diagnostic charting of panoramic radiography using deep-learning artificial intelligence system. Oral Radiol..

[61] Guler Ayyildiz B., Karakis R., Terzioglu B., Ozdemir D. (2024). Comparison of deep learning methods for the radiographic detection of patients with different periodontitis stages. Dentomaxillofac. Radiol..

